# Mexico’s Laboratory-Confirmed Human Case of Infection with the Influenza A(H5N2) Virus

**DOI:** 10.3390/v17020205

**Published:** 2025-01-31

**Authors:** Joel Armando Vázquez-Pérez, Claudia Wong-Arámbula, Mario Solís-Hernández, Eduardo Becerril-Vargas, Gisela Barrera-Badillo, Víctor Hugo Ahumada-Topete, Santiago Avila-Rios, Rogelio Pérez-Padilla, Fidencio Mejía-Nepomuceno, Enrique Mendoza-Ramírez, Marisol Karina Rocha-Martinez, Carlos Javier Alcazar-Ramiro, Alfredo Cruz, Joaquin Zúñiga, Karolina Bozena Piekarska, Dayanira Sarith Arellano-Suarez, María Natividad Cruz-Ortiz, Tatiana Ernestina Núñez-García, Eréndira Molina-Gómez, Laura Adriana Flores-Cisneros, Rodrigo Aparicio-Antonio, Abril Rodríguez-Maldonado, Magaly Landa-Flores, Armando García-López, Jorge Membrillo-Hernández, Gabriel García-Rodríguez, Herlinda García-Lozano, Irma López-Martínez, Ruth Purisima González-Sánchez, Carmen Margarita Hernández-Cárdenas

**Affiliations:** 1 Instituto Nacional de Enfermedades Respiratorias, “Ismael Cosío Villegas” Secretaría de Salud, Mexico City 14080, Mexico; joevazpe@gmail.com (J.A.V.-P.); edobec.var@gmail.com (E.B.-V.); victor.ahumada@uehi.mx (V.H.A.-T.); santiago.avila@cieni.org.mx (S.A.-R.); perezpad@gmail.com (R.P.-P.); biolfimene@gmail.com (F.M.-N.); heinrichunam@gmail.com (E.M.-R.); alfredocl@gmail.com (A.C.); joazu@yahoo.com (J.Z.);; 2 Instituto de Diagnóstico y Referencia Epidemiológicos, Dirección General de Epidemiología, Secretaría de Salud, Mexico City 01480, Mexicogisela.barrera20@yahoo.com.mx (G.B.-B.); esque_d@hotmail.com (D.S.A.-S.); natividad.cruz@gmail.com (M.N.C.-O.); nugtt@outlook.com (T.E.N.-G.); erendira.molina@salud.gob.mx (E.M.-G.); rodrigo.aparicio@salud.gob.mx (R.A.-A.); abril.rodriguez@salud.gob.mx (A.R.-M.); magaly.landa@salud.gob.mx (M.L.-F.); jorge.membrillo@salud.gob.mx (J.M.-H.); hgarcialozano@yahoo.com.mx (H.G.-L.); lopezmi74@gmail.com (I.L.-M.); 3 Comisión México-Estados Unidos para la Prevención de la Fiebre Aftosa y otras Enfermedades Exóticas de los Animales (CPA), SENASICA, Mexico City 05110, Mexico; mario.solis@senasica.gob.mx (M.S.-H.); carlos.alcazar@senasica.gob.mx (C.J.A.-R.); armando.garcia@senasica.gob.mx (A.G.-L.); 4 Centro Nacional de Referencia en Parasitología Animal y Tecnología Analítica (CENAPA), SENASICA, Mexico City 62550, Mexico; marisol.rocha@senasica.gob.mx; 5 Tecnologico de Monterrey, Escuela de Medicina y Ciencias de la Salud, Mexico City 14380, Mexico; 6 Dirección General de Epidemiología, Secretaría de Salud, Mexico City 01480, Mexico; lauraflores.cisneros@gmail.com (L.A.F.-C.); gabriel.garciar@salud.gob.mx (G.G.-R.); ruth.gonzalezs@salud.gob.mx (R.P.G.-S.)

**Keywords:** influenza, H5N2, RT-PCR, avian virus, Mexico

## Abstract

In April 2024, the Instituto Nacional de Enfermedades Respiratorias of Mexico City identified a case of unsubtypeable Influenza A in a 58-year-old immunocompromised patient with renal failure due to diabetic nephropathy and bacterial peritonitis. Through sequencing the M, NS, NA, NP, and HA complete segments, we identified an H5N2 influenza virus with identity of 99% with avian influenza A(H5N2) from Texcoco, Mexico, in 2024. This case is the first reported with direct evidence of human infection caused by the H5N2 influenza virus; the relationship of the virus with the severity of his condition remains unknown.

## 1. Introduction

Human infections with avian influenza viruses (AIVs) have been reported since the 1990s [1]. The main subtypes causing occasional respiratory diseases in humans are H5N1, H7N9, and H9N2 [2]. Additionally, H5N1 or H7N3 viruses were recently discovered in patients from North America who had mild symptoms such as conjunctivitis but no respiratory system involvement [3,4]. Furthermore, the avian H5N2 subtype is primarily seen in migratory birds and commercial and backyard poultry [5]. The first avian influenza H5N2 strain was initially described as infecting chickens in Pennsylvania in April 1983 [6]. To date, viral clades of H5N2 have been described in several countries, including Mexico, infecting chickens and other bird species [5]. 

In 1994, Mexican health authorities reported cases of low-pathogenicity H5N2 infections in chickens. However, at the end of 1994, highly pathogenic H5N2 strains were detected on several poultry farms, causing serious economic consequences amongst poultry farmers [6]. 

In March 2024, a high-pathogenicity H5N2 outbreak was identified on a poultry farm in Michoacán State. During the same month, two low-pathogenicity H5N2 outbreaks were reported in backyard poultry in the State of Mexico, in the municipalities of Texcoco and Temascalapa [7]. 

Some serological reports suggest potential subclinical H5N2 infections that were not detected [8,9]. However, to our knowledge, no human H5N2 infection has previously been documented. We present a case of respiratory sickness caused by the avian influenza H5N2 subtype in a patient who was admitted to the National Institute for Respiratory Diseases (INER) in Mexico City in April 2024.

## 2. Case Description

### 2.1. Clinical Presentation and Epidemiology

A male of 58 years of age was admitted to the emergency room at the Instituto Nacional de Enfermedades Respiratorias (INER), Mexico City, experiencing low back pain, diarrhea, nausea, and the low oxygenation rate of 80%. At admission, he had a SaO_2_ of 77.8% and was quickly placed in invasive mechanical ventilation and treated with volume replacement, vasopressor, and bicarbonate for shock and severe acidosis. His laboratory tests (Supplemental Table S1) showed leukopenia, neutrophilia, lymphopenia, moderate microcytic, hypochromic, anemia, and mild thrombocytopenia. Renal function was severely impaired, with severe azotemia and hyperglycemia, coagulation test alterations with prolonged times, elevated D-dimer, fibrinogen, myoglobin, high-sensitivity troponin, and elevated procalcitonin. A bilateral pleural effusion was observed on his chest X-ray. He presented with type 2 diabetes mellitus with poor metabolic control and end-stage chronic renal failure secondary to diabetic nephropathy and had been on peritoneal dialysis since the past year. The clinical diagnosis was septic shock due to bacterial peritonitis. The patient continued with hemodynamic deterioration, presenting with irreversible shock in response to vasopressors, worsening refractory metabolic acidosis with pH 6.9, and severe hyperlactatemia, finally dying 20 h after his admission to the emergency room. As a part of a routine clinical sampling, a respiratory sample was typed using specific primers for influenza H3N2 and B and A/H1N1pdm09 according to the Center of Disease Control (CDC) guidelines, and the influenza A unsubtypeable was detected. The Molecular Biology of Emerging Diseases Laboratory at the INER reported the presence of H5N2 characterized by sequencing after confirmation by the Instituto de Diagnostico y Referencia Epidemiologicos (InDRE) via the sequencing of the M segment.

The epidemiological investigation included communication with household contacts and an interview with his partner, who denied the presence of respiratory symptoms, living with animals, contact with birds, or any poultry-related activities. Seventeen suspected cases were identified, including contacts at the hospital and family members. Other surveillance activities included intentionally searching for suspected cases of zoonotic influenza across the two blocks around the home of the confirmed case. No identified contact was confirmed to have influenza H5N2.

#### H5N2 Molecular Characterization and Phylogenetic Analyses

The eight viral genome segments were amplified simultaneously from the clinical sample, and libraries for the eight segments were sequenced on a MiSeq platform (Illumina, San Diego, CA, USA). The DRAGEN COVIDSeq Targeted Microbial Pipeline on BaseSpace Sequence Hub was used for analysis, mapping, and consensus sequence obtaining. 

Using a de novo assembly pipeline of 11,139 unique influenza-specific reads, we obtained five complete segments: NS, M, NA, NP, and HA. (Supplementary Materials). The identity of the influenza viral consensus was determined, showing 97–98% with sequences of avian H5N2 from 2019 of Central Mexico (Influenza A virus (A/chicken/Queretaro/CPA-04673-1/2019(H5N2)).

**Figure 1:**
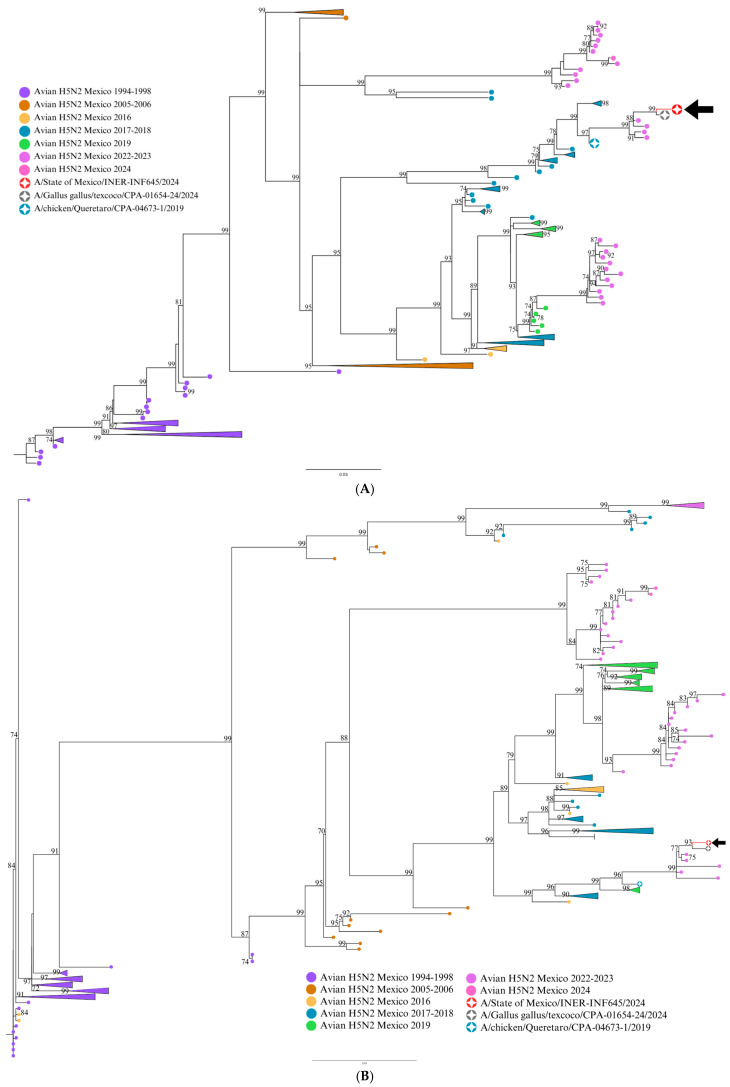
Maximum likelihood (ML) phylogenetic trees for HA (**A**) and NA (**B**) influenza H5N2 genetic segments. ML trees from 222 avian influenza H5N2 viruses (LPAIVs) registered in GenBank were produced with 1000 bootstrap replicates. The 2024 human sequence from Mexico is included (red circle and black arrow), along with sequences from Queretaro 2019 (green circle) and Texcoco 2024 (gray circle). Avian sequences from different years are indicated with colored circles. Bootstrap values higher than 70% are indicated. The scale bar indicates the nucleotide substitutions per site.

**Figure 2:**
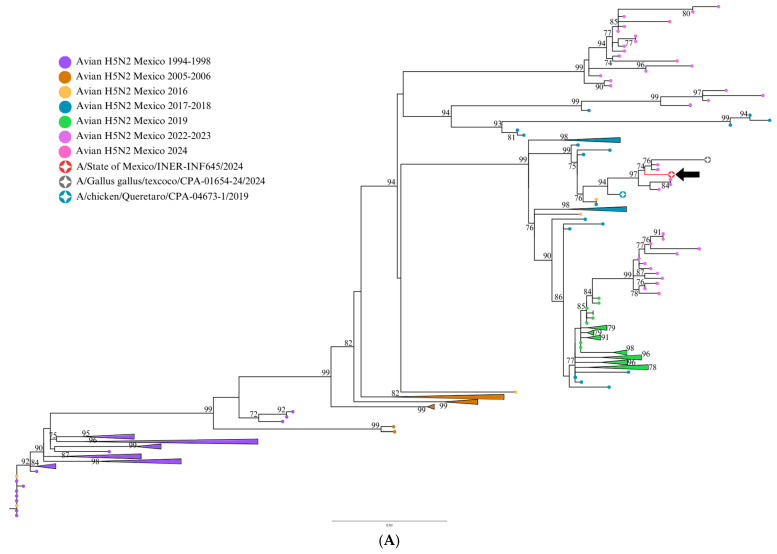

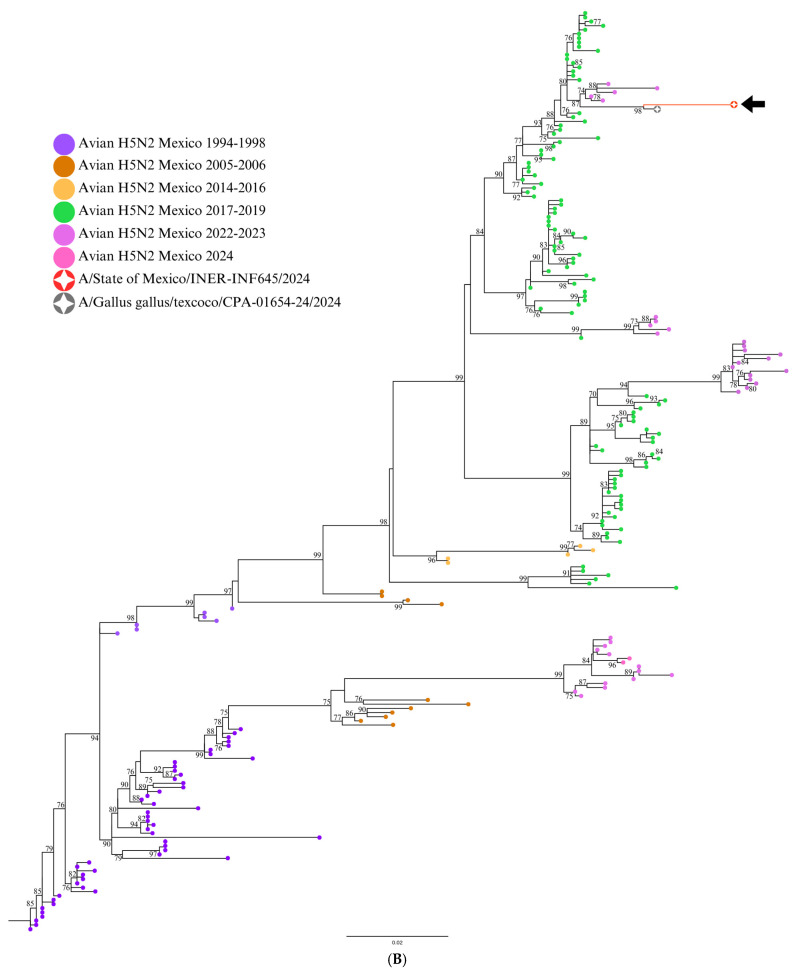
Maximum likelihood (ML) phylogenetic trees for M (**A**) and NS (**B**) influenza H5N2 genetic segments. ML trees from 222 avian influenza H5N2 viruses (LPAIVs) registered in GenBank were produced with 1000 bootstrap replicates. The 2024 human sequence from Mexico is included (red circle and black arrow), along with sequences from Queretaro 2019 (green circle) and Texcoco 2024 (gray circle). Avian sequences from different years are indicated with colored circles. Bootstrap values higher than 70% are indicated. The scale bar indicates the nucleotide substitutions per site.

**Figure 3:**
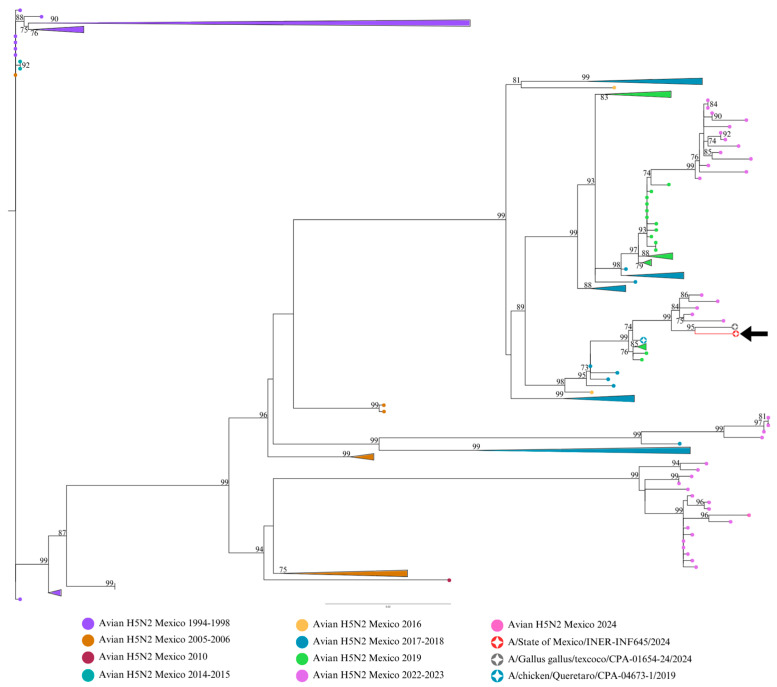
Maximum likelihood (ML) phylogenetic trees for NP influenza H5N2 genetic segments. ML trees from 222 avian influenza H5N2 viruses (LPAIVs) registered in GenBank were produced with 1000 bootstrap replicates. The 2024 human sequence from Mexico is included (red circle and black arrow), along with sequences from Queretaro 2019 (green circle) and Texcoco 2024 (gray circle). Avian sequences from different years are indicated with colored circles. Bootstrap values higher than 70% are indicated. The scale bar indicates the nucleotide substitutions per site.

Phylogenetic analysis showed the sequences of segments M, NA, NP, and HA clustered with the sequence from 2019 and with sequences from 2022, 2023, and 2024 (Figure 1, Figure 2 and Figure 3). The closest identity (99%) was for a 2024 avian sequence from State of Mexico. This virus is related to the high disease burden and mortality in backyard farms in Texcoco, a locality in the State of Mexico near the residence of the patient in the present study. Moreover, there were only three non-synonymous mutation differences between the two isolates: E489A in HA, G114R in NS1, and T433P in NP. The multi-basic amino acids at the cleavage site in HA showed four basic amino acids: PQKRKR/G. According to previous studies [5], the observed cleavage site sequence would classify the studied virus as a low-pathogenicity isolate (LPAIV). E119 V/D/Q, H274Y, R292K, and N294S in NA, which confer resistance to oseltamivir, zanamivir, and peramivir in H5N2 viruses, were not present in this isolate. HA molecular markers associated with increased binding to human-type receptors such as E190N, Q226L, and G128S were not present in this isolate.

Molecular evidence suggests that the human isolate in this study (INER_INF645_24) and the avian isolates from 2022, 2023, and 2024 possibly derive from a common avian H5N2 ancestor from 2019 from Central Mexico. The highest homology (99%) of the study virus being with an avian H5N2 isolate from Texcoco, State of Mexico (2024), suggests a direct relationship between these isolates. Moreover, molecular evidence suggests that the human isolate in this study and the avian isolates from 2022, 2023, and 2024 possibly derive from a common avian H5N2 ancestor from 2019 from Central Mexico. It is plausible that the avian virus causing a high disease burden in chickens in this geographical area in 2024 could be the source of the human case described here. 

## 3. Discussion

The detection and molecular characterization of influenza virus H5N2 in a respiratory sample confirmed the first report of human infection due of this subtype in Mexico. 

Molecular evidence suggests that the human isolate of this study (INER_INF645_24) and the avian isolates from 2022, 2023, and 2024 possibly derive from a common avian H5N2 ancestor from 2019 from Central Mexico (Influenza A virus (A/chicken/Queretaro/CPA-04673-1/2019(H5N2)). The observation of the highest homology (99%) of the study virus being with an avian H5N2 isolate from Texcoco, State of Mexico (2024), suggests a direct relationship between these isolates. Although direct contact between the patient in this study and poultry or other domestic animals could not be confirmed, it is plausible that this avian virus causing high disease burden in chickens in this geographical area in 2024 could be the source of the human case described here, as human-to-human transmission seems unplausible. 

This is the first report of a human case of influenza H5N2 infection in Mexico. Further studies are required to determine the predicted pathogenicity of the virus and to predict its capability of human-to-human transmission and potential threat to human health. Unfortunately, several comorbidities in the case described here led to a fatal outcome, but the pathogenicity of the isolate needs to be further studied. 

Since no cases of H5N2 influenza in humans have been reported so far, we are unaware of the clinical outcomes that this influenza virus subtype may have in humans. At admission, the patient was severely ill, with renal failure and bacteremic infection. It is uncertain what contribution the influenza virus H5N2 made to the final clinical status of the patient, and it is also unknown how the patient acquired the influenza virus, which is very similar to bird viruses identified in the Valley of Mexico in 2024. 

## Data Availability

The genomic information generated during the current study is available in GenBank accession no. PP886231-PP886235 and GISAID EPI3358335-39. Sequences of avian influenza of 2024 from Mexico were deposited in GenBank under accession no PP929863-PP929894.

## Supplementary Materials

The following supporting information can be downloaded at: https://www.mdpi.com/article/10.3390/v17020205/s1, Figure S1: Avian H5N2 outbreaks in Mexico in 2023–2024. Colored states indicate where Avian H5N2 outbreaks occurred. The black arrow indicate Texcoco, State of Mexico, where the H5N2 human case occurred. Table S1: Clinical laboratory tests of the patient. Sequencing methodology and references. Refs. [10,11,12] are cited in Supplementary Materials.
