# Supplementary material for: Mexico’s Laboratory-Confirmed Human Case of Infection with the Influenza A(H5N2) Virus
Source: Viruses. 2025 Jan 31;17(2):205. doi: 10.3390/v17020205 (PMC11861177; doi:10.3390/v17020205)
Supplement: Supplementary file 1 [file viruses-17-00205-s001.zip › viruses-3432064-supplementary.pdf]

Supplemental material

Table S1. Clinical Laboratory Results

Variable	Patient Values	Reference Values
**Hemoglobin (g/dl)**	8.9	15.6-18.5
**Hematocrit (%)**	28.7	46-54
**Leukocytes (10^3 mm^3)**	3740	4.9–10.9
**Differential Count**		
**Neutrophils (10^3 mm^3)**	3330	1.7-7.6
**Lymphocytes (10^3 mm^3)**	220	1-3.2
**Monocytes (10^3 mm^3)**	190	0.3-1.1
**Eosinophils (10^3 mm^3)**	0	0-0.5
**Platelets (10^3 mm^3)**	114000	175-388
**Glucose (mg/dl)**	274	70-99
**Creatinine (mg/dl)**	14.5	0.77-1.32
**Blood Urea Nitrogen (mg/dl)**	174	7-25
**Urea (mg/dl)**	372	15-58
**Potassium (mmol/L)**	3.3	3.7-5.2
**Sodium (mmol/L)**	141	136-145
**Chloride (mmol/L)**	104	102-112
**Albumin (g/dl)**	1.44	3.5-5.7
**Total Bilirubin (mg/dl)**	0.74	0.22-1.04
**Alanine aminotransferase (U/L)**	15	13-39
**Aspartate aminontransferase (U/L)**	9	9-47
**Lactate dehydrogenase (U/L)**	202	140-271
**Protrombin time (seconds)**	14	9.4-12.5
**Activated partial thromboplastin time (seconds)**	47.2	25.1-36.5
**International Normalizated Ratio**	1.28	0.7-1.24
**D-dimer (ng/ml)**	5251	0-500
**Fibrinogen (mg/dl)**	321	238-498
**B-Type Natriuretic Peptide (pg/ml)**	292	<100
**Myoglobin (ng/ml)**	713.2	0-154
**Troponin (pg/ml)**	63.8	0-34.2
**Procalcitonin (ng/ml)**	20.85	<0.08

Sequencing Methodology

Demographic data, clinical symptoms, laboratory data, and outcome-related information were obtained by electronic medical records. Clinical management was performed according to the standard of care and clinical criteria by attending physicians.

RNA extraction and sequencing

Viral RNA was extracted from 200 μL of nasopharyngeal swabs (sample INER_INF645_24), using QIAamp Viral RNA mini kit (QIAGEN). The 8 viral genome segments were amplified simultaneously and directly from the clinical sample, using MBTuni12 and MBTuni13 primers, as described elsewhere (1-4). Libraries for the 8 viral segments were generated using the reagents of the Covid-Seq kit (Illumina, San Diego, CA, USA). Libraries were sequenced on a MiSeq platform using a 2 x 150-cycle to obtain paired-end reads (Illumina, San Diego, CA, USA). The DRAGEN COVIDSeq Targeted Microbial Pipeline on BaseSpace Sequence Hub was used for the analysis, mapping, and consensus sequence obtention. MEGAHIT was used to perform de novo assembly on the scrubbed reads. CD-HIT-EST was used to cluster similar contigs to reduce redundancy. The resulting contigs were mapped to a set of reference genomes using minimap2.

Sequences of five genome segments (NS, M, NA, NP and HA) with coverage depth 834X, 773X, 43X, 33X and 50X respectively of the sample INER_INF645_24 were deposited in GenBank under accession no. PP886231 - PP886235

and GISAID EPI3358335-39. Sequences of avian influenza of 2024 from Mexico were deposited in GenBank under accession no PP929863- PP929894. Blast online https://blast.ncbi.nlm.nih.gov/Blast.cgi was used to assess the identities of viral consensus sequences.

Phylogenetic analysis

To perform phylogenetic analysis, we analyzed 222 complete genome sequences of avian influenza H5N2 available on the GenBank platform from different states in Mexico (1994-2024). Sequence alignments were created using MAFFT V7 (1) and edited with MEGA 10.0 (2). A maximum likelihood tree was constructed for the whole genome sequence using MEGA 10.0. The General Time-Reversible model was selected with five-parameter gamma-distributed rates and 1000 bootstrap replicates. Edition of the trees was made using FigTree (3).

References

1. Katoh, K.; Misawa, K.; Kuma K ichi Miyata, T. MAFFT: a novel method for rapid multiple sequence alignment based on fast Fourier transform. *Nucleic Acids Res* 15 de julio de 2002;30(14):3059-66.

2. Kumar S, Stecher G, Li M, Knyaz C, Tamura K. MEGA X: Molecular Evolutionary Genetics Analysis across Computing Platforms. Mol Biol Evol. 1 de junio de 2018;35(6):1547-9.

3. Rambaut A. FigTree [Internet]. [citado 13 de marzo de 2024]. Disponible en: http://tree.bio.ed.ac.uk/software/figtree/
